# DISPARITIES IN DEPRESSION AMONG CHINESE OLDER ADULTS

**DOI:** 10.1093/geroni/igz038.3222

**Published:** 2019-11-08

**Authors:** Zhichao Hao, qingyi Li, Nicole Ruggiano

**Affiliations:** University of Alabama, Tuscaloosa, Alabama, United States

## Abstract

Depression has become one of the major health issues among older adults. In turn, various factors can facilitate or impede the occurrence of depression, socially, economically and culturally. Around 2050, China will have 487 million older adults or nearly 35 percent of the total population. Due to the different background of society, economy and culture, what explanations and knowledge can China provide based on present experiences and practices to help better understand depression among older adults from more comprehensive way? This study was conducted on the latest wave (2011-2014) of the Chinese Longitudinal Healthy Longevity Survey (CLHLS, 1998-2014). The sample included 7,107 Chinese older adults age from 65 to 117 years in China. A binomial hierarchical logistic regression was performed to examine the likelihood of having depression among older adults predicted by geographic characteristics, quality of life, chronic diseases, personal community services, social community services, and demographic variables including gender, age, and current marital status. Analysis indicated that approximately 10% of Chinese older adults in the sample reported depression. Compared to female and young-old adults (age 65-74), males (OR=.636, p<.001) and oldest-old adults (age 95+) (OR=.822, p<.001) were less likely to have depression. Older adults who lived in rural areas (OR=.681, p<.001) showed less likelihood of having depression. Older adults who had better life quality (OR=.553, p<.001) revealed less likely to have depression. Having social services in the community (OR=.908, p<.05) significantly lowered the likelihood of having depression among Chinese older adults. Implications for research, policy, and practice are discussed.

